# Service Delivery Models to Maximize Quality of Life for Older People at the End of Life: A Rapid Review

**DOI:** 10.1111/1468-0009.12373

**Published:** 2019-03-18

**Authors:** CATHERINE J. EVANS, LUCY ISON, CLARE ELLIS‐SMITH, CAROLINE NICHOLSON, ALESSIA COSTA, ADEJOKE O. OLUYASE, EVE NAMISANGO, ANNA E. BONE, LISA JANE BRIGHTON, DEOKHEE YI, SARAH COMBES, SABRINA BAJWAH, WEI GAO, RICHARD HARDING, PAUL ONG, IRENE J. HIGGINSON, MATTHEW MADDOCKS

**Affiliations:** ^1^ King's College London, Cicely Saunders Institute of Palliative Care Policy and Rehabilitation; ^2^ Sussex Community NHS Foundation Trust Brighton General Hospital; ^3^ King's College London, Florence Nightingale Faculty of Nursing Midwifery & Palliative Care; ^4^ St Christopher's Hospice; ^5^ World Health Organisation Centre for Health Development

**Keywords:** palliative care, geriatrics, health services for the aged, quality of life

## Abstract

**Policy Points**

- We identified two overarching classifications of integrated geriatric and palliative care to maximize older people's quality of life at the end of life. Both are oriented to person‐centered care, but with differing emphasis on either function or symptoms and concerns.
- Policymakers should both improve access to palliative care beyond just the last months of life and increase geriatric care provision to maintain and optimize function. This would ensure that continuity and coordination for potentially complex care needs across the continuum of late life would be maintained, where the demarcation of boundaries between healthy aging and healthy dying become increasingly blurred.
- Our findings highlight the urgent need for health system change to improve end‐of‐life care as part of universal health coverage. The use of health services should be informed by the likelihood of benefits and intended outcomes rather than on prognosis.

**Context:**

In an era of unprecedented global aging, a key priority is to align health and social services for older populations in order to support the dual priorities of living well while adapting to a gradual decline in function. We aimed to provide a comprehensive synthesis of evidence regarding service delivery models that optimize the quality of life (QoL) for older people at the end of life across health, social, and welfare services worldwide.

**Methods:**

We conducted a rapid scoping review of systematic reviews. We searched MEDLINE, CINAHL, EMBASE, and CDSR databases from 2000 to 2017 for reviews reporting the effectiveness of service models aimed at optimizing QoL for older people, more than 50% of whom were older than 60 and in the last one or two years of life. We assessed the quality of these included reviews using AMSTAR and synthesized the findings narratively.

**Results:**

Of the 2,238 reviews identified, we included 72, with 20 reporting meta‐analysis. Although all the World Health Organization (WHO) regions were represented, most of the reviews reported data from the Americas (52 of 72), Europe (46 of 72), and/or the Western Pacific (28 of 72). We identified two overarching classifications of service models but with different target outcomes: Integrated Geriatric Care, emphasizing physical function, and Integrated Palliative Care, focusing mainly on symptoms and concerns. Areas of synergy across the overarching classifications included person‐centered care, education, and a multiprofessional workforce. The reviews assessed 117 separate outcomes. A meta‐analysis demonstrated effectiveness for both classifications on QoL, including symptoms such as pain, depression, and psychological well‐being. Economic analysis and its implications were poorly considered.

**Conclusions:**

Despite their different target outcomes, those service models classified as Integrated Geriatric Care or Integrated Palliative Care were effective in improving QoL for older people nearing the end of life. Both approaches highlight the imperative for integrating services across the care continuum, with service involvement triggered by the patient's needs and likelihood of benefits. To inform the sustainability of health system change we encourage economic analyses that span health and social care and examine all sources of finance to understand contextual inequalities.

The world's population is aging, with an unprecedented rise in the number of people aged 60 years and older, and with the largest proportional increase being the oldest old.^1^, ^2^ With advancing age comes multimorbidity and frailty,^3^ as well as a prolonged and uncertain trajectory of functional decline that lasts years rather than months. Health and social care needs among older people are diverse and often complex, with multiple interacting factors related to the individual (eg, ethnicity), his or her health (eg, morbidities), and environment (eg, care setting, resources). Healthy aging means supporting the duality of living as well as possible by maximizing function and preventing or minimizing complications while adapting successfully to gradual deterioration and human finitude.^4^ It extends beyond longevity, particularly in the last years of life. The amelioration of suffering and concerns is imperative for older people, who often experience a high prevalence and level of symptom distress associated with advanced illness.^5^ However, there is often uncertainty as to when an older person is nearing the end of life and could benefit from a palliative approach, particularly those who are frail and have nonmalignant conditions, such as dementia. Accordingly, our recognition of the likely benefit of palliative and end‐of‐life care (EoLC) is frequently limited to the last days or weeks of life.^6^ This late recognition can impede care, with overuse of aggressive treatments that have little benefit and compromise quality of life^7^ and undertreatment of symptoms and concerns, notably or including pain, anxiety, and breathlessness.^8^ Health and social care providers’ poor communication regarding the goals and plans of care, as well as limited involvement of the older person in decision making, further diminishes the intended benefit of the care provided.^9^

Palliative care is considered internationally as an essential health service for all people with chronic progressive conditions,^10^ and it is a key part of the required global systemwide response to realign health and social care to the needs of our aging populations.^1^ In 2014, the World Health Assembly (WHA)^11^ resolved that palliative care be integrated into all health systems. The WHA conceptualized palliative care as relevant across the illness trajectory, encompassing EoLC, delivered by all those providing care to people living and dying with chronic progressive conditions, and with the shared goals of improving quality of life and enabling people to die peacefully. The purpose of palliative care is to improve quality of life by preventing and/or relieving suffering through the early identification and impeccable assessment and treatment of physical, psychological, and spiritual symptoms and concerns for the person and his or her family.^11^, ^12^

Globally, there are many service delivery models for palliative and EoLC care for older people, spanning a specific condition (eg, dementia),^13^ care setting (eg, care homes),^14^ and provision by both specialist teams in palliative care for people with complex problems and generalists in palliative care treating those with progressive conditions,^15^ including primary and geriatric care.^16^, ^17^, ^18^ Most models advocate a comprehensive assessment, with an emphasis on supporting functional and mental capabilities and enabling the pursuit of those things important to the individual. Care delivery is by multidisciplinary teams, with an increasing understanding that optimal care requires the integration of services across health and social care systems.^19^ Low‐ and middle‐income countries (LMICs) rely particularly on community‐ and home‐based delivery,^20^, ^21^ and access to service is often confined to large urban centers.^22^ The global inequity in the provision of palliative care^15^ makes it imperative to identify models of care that are sustainable in LMICs. The health system challenges may be different; for example, the availability of analgesia is a pressing policy issue for many LMICs,^23^, ^24^, ^25^ and aging is a relatively new and rapidly changing epidemiological phenomenon.^1^ The WHO member states’ commitment to universal health coverage (UHC) by 2030 provides an opportunity to broaden access to care for older people with chronic progressive conditions and to realign health systems with the needs of our aging population. We must understand what the “best” systems and models of service delivery are and how to realign care to meet the complex health needs associated with advanced age across low‐, middle‐, and high‐income settings in order to achieve UHC.

We aimed to provide a comprehensive systematic synthesis of the available evidence regarding service delivery models that optimize quality of life for older people at the end of life. In response to a call from the WHO, we studied health, social, and welfare services across all countries, with special attention to LMICs. Our objectives were to (1) describe the context, components, and target outcomes of the overarching service delivery models; (2) summarize their reported impacts on quality of life, function, and dignity; (3) appraise the scalability and sustainability of service delivery models with respect to implementation requirements, workforce implications, and population coverage; and (4) identify priorities for policy, practice, and research.

## Methods

### Design

We conducted a rapid scoping review to systematically search, select, and synthesize knowledge regarding our aims to map key concepts, types of evidence, and gaps in research.^26^ We planned the review in accordance with the Centre for Reviews and Dissemination^27^ guidance and reported it in accordance with the Preferred Reporting Items for Systematic Reviews and Meta‐Analysis (PRISMA) statement.^28^

### Eligibility Criteria

We included systematic reviews published between January 2000 and October 2017 that examined service delivery models aimed at maximizing older people's quality of life at the end of life. Our primary intention was to assess the evidence for current models.

Operationally, we defined reviews of older people as those where 50% or more of the included population were 60 or more years old, and were at the end of life; that is, those people described as being in the last one to two years of life, using a service typically accessed during an advanced stage of disease (eg, specialist palliative care, nursing home) or having advanced disease. We defined a service delivery model as “an overarching design for health care service provision with multiple components and interacting elements.”^29^ Delivery models focusing on postdeath intervention were outside the scope of the review, and we excluded reviews of a single component intervention, for example, the provision of an assistive device. We did include reviews containing data on outcomes relating to quality of life, function, or dignified end‐of‐life care. Eligible reviews had to draw on more than one data source^30^ in order to identify studies using experimental designs. Narrative reviews, or those describing case studies or series or descriptive studies only, were not eligible.

### Search Strategy

We devised an electronic search strategy with an information specialist using a combination of MeSH and full‐text search terms developed for MEDLINE and adapted for other databases as necessary. The MeSH terms included “Terminally ill” or “Palliative care” for the population, “Hospice and palliative care nursing” or “Hospice care” for the intervention, and “Quality of life,” “Pain management,” or “Activities of daily living” for outcomes of interest. The Boolean operators OR and AND were used for MeSH terms within and across the population, interventions, and outcomes of interest. Key search terms were used as free text and also with the use of the truncation symbol to retrieve variations in the terminology (details in Online Appendix 1). We conducted electronic database searches in MEDLINE, CINAHL, EMBASE, and the Cochrane Database of Systematic Reviews. Our searches were restricted to human subjects and to systematic reviews using a filter developed by Lunny and colleagues.^31^ There was no restriction on the language of the publication. We searched the gray literature using hand searching; scanning reference lists, textbooks, and policy documents; and contacting experts in the field to seek potentially relevant research material, including ongoing and unpublished research.

#### Selection of Studies

We used referencing software (Endnote version x8)^32^ to manage a database of search findings and to remove duplicates. A calibration process took place in which two reviewers (Lucy Ison and Clare Ellis‐Smith) independently reviewed 50 random citations to test the application of the eligibility criteria. Once an agreement of more than 90% was confirmed, 4 groups of reviewers (Lucy Ison and Clare Ellis‐Smith; Catherine Evans, Deokhee Yi, and Lisa Jane Brighton; Anna Bone and Matthew Maddocks; Alessia Costa and Adejoke Oluyase) screened all the titles and abstracts. For titles and/or abstracts that met the review criteria or when information in the title and abstract was insufficient to determine eligibility, we retrieved the full‐text articles. In cases of discrepancy, the project leaders (Catherine Evans and Matthew Maddocks) appraised the full texts, along with a group discussion.

#### Assessment of Methodological Quality in Included Studies

Those reviews selected for inclusion were assessed for methodological quality using A Measurement Tool to Assess Systematic Reviews (AMSTAR),^33^ which has demonstrated satisfactory reliability and construct validity.^34^ AMSTAR covers 11 key constructs with a point allocated for the presence of each criterion (unweighted), with a maximum score of 11. Quality was categorized as low (scoring 0‐4), moderate (scoring 5‐8), or high (scoring 9‐11).^35^

#### Data extraction and Analysis

We developed and piloted a standardized data extraction form that contained data on the countries and health care systems represented by our review's primary studies, models of care, target population, outcome measures, impact on clinical and cost outcomes, stated limitations, scalability and sustainability, and implications for future research. The literature was summarized by respective WHO region and World Bank Classification income status, and the service delivery models were understood using the CATWOE Checklist (Customers, Actors, Transformation processes, World view, Owner, External influences).^36^, ^37^ Because we anticipated heterogeneity around the type of data and level of detail, rather than identify distinct model types with descriptions of how components interact in each model, we synthesized the analysis into three themes: (1) overarching service models, (2) service delivery context, and (3) common model components. Target outcomes were summarized by frequency counts. To assess the impact of service delivery models, we presented all quantitative statistics from meta‐analyses and categorized narrative syntheses as effective, inconsistent, not effective, or harmful. We summarized the health economic data according to the frequency of reporting, the types of costs measured, and the perspectives used (patient, hospital, health care system, society) to determine which costs and health benefits were being considered.

We assessed the service delivery models for level of coverage for LMICs, health systems, and the included populations’ political and sociocultural diversity. We appraised qualitatively the reviews including studies from LMICs for the extent to which they were inclusive of the health system structures and challenges such as palliative care development or cultural contexts, population characteristics, and disease epidemiology.^38^ Last, we synthesized narratively the implications of findings for policy and for areas for further research, stratifying them by level of economic development.

## Results

### Study Retrieval

**Figure 1:**
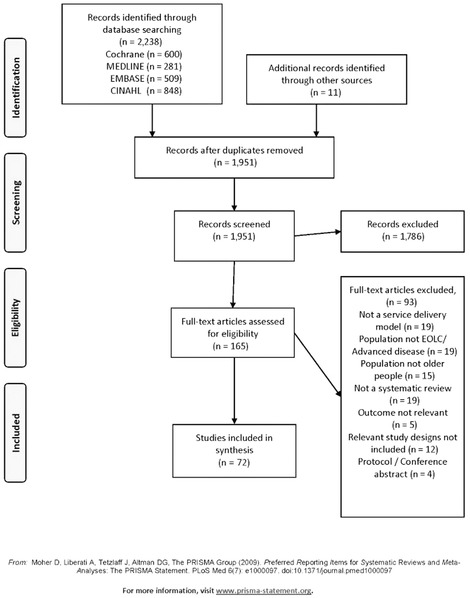
PRISMA Flow Diagram

Our search retrieved 2,238 articles. After removing duplicates and screening titles and abstracts, we retrieved 165 full‐text articles for further appraisal, of which 72 articles were eligible to be retained (Figure 1). Table 1 is an overview of these 72 reviews. An increasing number of reviews were published over time (2000‐2009, *n* = 17; 2010‐onward, *n* = 56) and the majority (49 of 72, 68%) limited their search strategy to articles published in 2000 or onward. All WHO regions were represented in the reviews, although the reviews predominantly included studies from the Americas (*n* = 52 of 72), European (*n* = 46 of 72), and the Western Pacific (*n* = 28 of 72) regions. Reviews including studies from other WHO regions and LMICs were less common; examples are Africa (Uganda,^39^ Kenya,^40^ Zambia, and South Africa^39^, ^41^), Southeast Asia (India, Nepal, and Pakistan^41^, ^42^), and the East Mediterranean (with studies from Israel^43^, ^44^, ^45^, ^46^, ^47^) (details in Online Appendix 3). Most of the reviews (71 of 72, 99%) contained effectiveness data, of which 20 of 72 (28%) reported meta‐analysis. Data from more than 784,983 individuals were available, and when stated, samples sizes ranged from 87 to 254,717 (see Table 1). The included reviews encompassed a breadth of service delivery models for older people nearing the end of life that intended to improve quality of life (see Online Appendix 2 detailing the aim for each included review).

**Table 1:** Overview of Included Systematic Reviews Categorized by Analysis Approach and Order by Number of Participants

	**WHO Region**						**World Bank Classification**						
	Americas	Europe	Eastern Med.	Western Pacific	Southeast Asia	Africa	High	Upper‐middle	Lower‐middle	Low	**No. of Participants**	**AMSTAR Quality Appraisal**	**No. of Studies**
**Meta‐analysis**													
Gomes et al. 2013^48^	x	x		x			x				41,603	10	23
Lowthian et al. 2015^49^	x	x		x			x				22,502	5	9
Diop et al. 2017^50^	x						x				20,105	8	15
Stuck et al. 2002^51^	x	x		x			x				13,447	6	18
Kavalieratos et al. 2016^40^	x	x		x		x	x		x		12,731	8	43
Ellis et al. 2011^52^	x	x		x			x				10,315	8	22
Fox et al. 2012^94^	x	x					x	x			6,839	9	13
McAlister et al. 2004^54^	x	x		x			x				6,320	4	29
Ekdahl et al. 2015^55^	x	x					x				6,005	6	17
Phillips et al. 2013^56^	x	x		x			x				3,304	8	18
De Coninck et al. 2017^57^	x			x			x				3,163	7	9
Health Quality Ontario 2014^58^	x	x		x			x				2,602	7	10
Conroy et al. 2011^47^											2,287	8	5
Haun et al. 2017^59^	x	x		x			x				1,614	9	7
Shepperd et al. 2016^60^	x	x					x				823	9	4
Brereton et al. 2017^36^							x				‐	9	18
Higginson and Evans 2010^61^	x	x		x			x				‐	6	40
Higginson et al. 2002^38^	x	x					x				‐	8	13
Higginson et al. 2003^62^	x	x					x				‐	9	44
Luckett et al. 2014^63^							x	x			‐	5	43
**Meta‐synthesis (Narrative Synthesis)**													
Candy et al. 2011^64^	x	x		x			x				25,4717	7	18
Kim and Tarn 2016^65^	x	x		x			x				95,006	5	13
Pillotto et al. 2017^18^											75,181	4	39
May et al. 2014^66^	x						x				40,069	4	10
Simoens et al. 2010^45^	x	x	x	x			x				30,647	6	15
Rizzo and Rowe 2016^67^	x						x				19,416	3	42
Young et al. 2017^68^	x	x		x			x				16,377	10	10
Oeseburg et al. 2009^69^	x	x					x				15,746	7	9
Easton et al. 2016^70^	x	x		x			x				11,852	6	19
El‐Jawahri et al. 2011^17^	x	x					x				10,596	3	22
You et al. 2012^44^	x	x		x			x	x			8,095	6	15
You et al. 2013^43^	x	x		x			x	x			8,095	5	21
Hopman et al. 2016^71^	x	x		x			x				7,946	6	19
Roczen et al. 2016^72^	x	x					x				7,629	4	12
Windham et al. 2003^73^	x	x		x			x				6,919	1	32
Richards and Coast 2003^74^	x	x		x			x				5,718	4	15
Dy et al. 2013^75^											5,666	6	23
Latour et al. 2007^76^											5,092	7	10
Zimmermann et al. 2008^77^	x	x					x				4,804	9	22
Puts et al. 2017^78^	x	x		x	x		x				3,632	8	14
Nevis 2014^79^	x	x					x				3,170	7	6
Bai et al. 2013^80^	x	x		x			x	x			3,092	4	18
Frank and Wilson 2015^81^	x						x				3,044	0	4
Pham and Krahn 2014^82^											3,009	3	6
Kane et al. 2015^83^	x	x					x				2,540	6	10
Eklund et al. 2009^84^	x	x					x				2,259	4	9
Garcia‐Perez 2009^85^		x					x				2,198	7	4
Martinez et al. 2014^86^	x	x		x			x				2,027	4	19
Ryburn et al. 2009^87^	x	x		x			x				1,782	4	3
Catania et al. 2015^88^	x	x					x				1,702	8	10
Nordly et al. 2016^89^		x		x			x				1,590	5	8
Carpenter 2017^90^	x	x		x			x				1,263	3	12
Candy et al. 2012^91^	x						x				1,130	8	5
Bakitas et al. 2015^41^	x	x		x	x	x	x	x	x		873	3	28
Hall et al. 2011^14^	x						x				735	11	3
Alcide and Potocky 2015^92^	x						x				449	6	5
Ruiz‐Iniguez et al. 2017^93^											343	6	8
Phillips et al. 2004^94^	x	x		x			x				293	7	9
Sampson et al. 2005^95^	x						x				263	6	3
Singh and Harding 2015^42^	x	x	x	x					x	x	148	5	16
Procter 2012^96^		x					x				87	5	5
Bainbridge et al. 2016^46^	x	x	x	x			x				‐	6	19
Bakker et al. 2011^97^	x	x					x				‐	6	20
Dy et al. 2008^98^											‐	0	72
Hodgkinson et al. 2011^99^	x	x					x				‐	10	2
Joseph et al. 2016^100^											‐	3	0
Lorenz et al. 2008^16^	x	x		x			x				‐	8	89
Lupari et al. 2011^101^	x	x					x				‐	6	8
Maharaj and Harding 2016^102^	x						x	x			‐	5	9
Robinson et al. 2009^39^	x	x		x		x	x	x			‐	5	5
Singer et al. 2016^103^												3	124
Soares et al. 2012^104^												3	33

### Quality Appraisal

The median methodological score was 6 (range 0‐11). Most reviews were categorized as moderate (42 of 72, 58%), or low (20 of 72, 28%), with only a few considered as high quality (10 of 72, 14%). The methods used to combine studies were appropriate in most of the reviews (63 of 72, 88%); 53 of 72 (74%) reviews used an a priori design; and 51 of 72 (71%) used a comprehensive literature search strategy. However, only 22 of 72 (31%) reviews detailed the status of publication in the inclusion criteria (eg, gray literature), and 21 of 72 (29%) included a conflict of interest and assessed the likelihood of publication bias (details in Online Appendix 4). Owing to the heterogeneity of studies, we made a post hoc decision not to use quality criteria in any sensitivity analysis.

#### Service Delivery Models

We identified two overarching classifications of service delivery models, “Integrated Geriatric Care” and “Integrated Palliative Care.” Both involved services working together to align care with the person's needs, concerns, and goals. The distinction between the models related to the timing in relation to functional decline, target outcomes, and leading care provider(s). The Integrated Geriatric Care model placed greater emphasis on outcomes to improve physical function and typically concerned earlier stages of functional decline, while Integrated Palliative Care tended to focus more on outcomes of symptom severity and psychosocial/spiritual concerns during the advanced disease stage up to death. Integrated Palliative Care encompassed both specialist palliative care services provided by teams for patients with complex problems, as well as generalist services from providers with a basic knowledge of palliative care and treating patients with chronic progressive conditions (Table 2).

**Table 2:** Typology of Service Delivery Models

**Overarching Service Delivery Models**	**Reviews (n)**	**References**
**Integrated Geriatric Care**	25	17, 40, 42, 43, 49, 50, 53, 54, 57, 59, 65 ^,^ 67, 68, 70, 71, 73, 75, 81, 83, 87, 88, 94 ^,^ 98, 104, 105
Person‐centered care involving services working together, mainly accessed at an earlier trajectory of functional decline, focusing on quality of life, with emphasis on maintaining function.		
**Integrated Palliative Care**	30	14, 17, 38, 39, 40, 41, 42, 43, 46, 48, 54, 58, 59, 60, 61, 62, 63, 66 ^,^ 67, 72, 73, 77, 82, 85, 89, 90, 96, 100 ^,^ 102, 103
Person‐centered care involving services working together, commonly accessed at a later trajectory of functional decline and dying, focusing on quality of life, with emphasis on reducing symptom distress and concerns.		
Specialist: for patients with complex problems (majority of service delivery models).		
Generalist: provided by those with basic knowledge of palliative care treating patients with life‐limiting conditions.		
**Overarching Methods to Integrate and Manage the Continuum of Care**		
**Comprehensive Assessment**	14	18, 40, 49, 51, 55, 67, 70, 73, 74, 77, 78 ^,^ 82, 87, 97
Person‐centered assessment of needs across physical, psychological, social, and spiritual domains.		
**Case Management**	30	17, 40, 43, 44, 47, 49, 51, 54, 56, 59 ^,^ 67, 68, 69, 70, 71, 73, 74, 76, 78, 84, 86, 87, 94, 97 ^,^ 98, 99, 101, 103, 104, 105
Coordinating care for patients and their caregivers by assigning each case to an individual and/or a team.		
**Collaborative Working**	41	17, 36, 39, 40, 42, 43, 49, 50, 54, 55 ^,^ 57, 58, 59, 61, 62, 65, 66, 67, 68, 69, 70, 71, 72, 73, 75, 77, 78, 80 ^,^ 81, 83, 84, 85, 87, 94, 96, 97, 98, 100, 104, 105
Working across disciplines and organizations to plan and deliver services to meet the needs of individuals and those close to them.		
**Shared Key Components and Subcategories**		
**Person‐Centered Care**		
Multi‐ or single‐component (physical, psychosocial, and spiritual)		
1. Person (physical, psychosocial, spiritual)	62	17, 39, 40, 41, 42, 44, 46, 48, 53, 54, 55, 56, 57, 58, 60, 61 ^,^ 63, 65, 68, 70, 71, 73, 78, 80, 81, 86, 87 ^,^ 92, 94, 97, 98, 101, 103, 105
2. Person (physical, eg, symptom management)	33	17, 36, 40, 43, 46, 48, 49, 53, 54, 55, 59 ^,^ 60, 62, 63, 66, 67, 68, 69, 71, 72, 73, 80, 81, 83, 85 ^,^ 87, 94, 96, 97, 98, 100, 103
3. Person (psychosocial)	28	36, 40, 43, 44, 46, 48, 49, 53, 54, 55, 56, 59 ^,^ 60, 62, 66, 67, 69, 71, 72, 73, 75, 77, 80, 83 ^,^ 85, 97, 100, 104
4. Person (spiritual)	5	40, 54, 80, 85
5. Person (unspecified)	4	14, 46, 78, 82
**Education**		
Education (patients and/or caregivers; staff)	23	14, 17, 40, 41, 42, 43, 48, 52, 54, 55, 60, 61 ^,^ 67, 71, 73, 79, 80, 83, 86, 87, 92, 103, 104
Education (patients and/or caregivers)	18	17, 40, 41, 43, 48, 54, 55, 71, 73, 79 ^,^ 80, 86, 87, 92, 103, 104, 105
Education (staff)	11	14, 40, 41, 42, 60, 61, 67, 79, 83, 86, 104
**Workforce**	10	14, 40, 41, 46, 52, 58, 61, 70, 97, 104

We identified three common methods to integrate care across services and manage the continuum of care over time: Comprehensive Assessment (discussed in general terms or as a specific model, eg, the Comprehensive Geriatric Assessment)^52^, ^55^; Case Management; and Collaborative Working (see Table 2). Those components of the service delivery model shared across the two overarching classifications were person‐centered care, education, and workforce. Person‐centered care was the component found most often. This care mainly focused on physical and psychosocial interventions, with less consideration of spiritual concerns. Education was subdivided into its recipients: patient and/or caregiver with training on, for example, self‐management of chronic illness^71^; and staff with training on, for example, palliative care by specialist palliative care teams^61^ or workforce development programs to support EoLC in nursing homes.^14^

#### Service Delivery Context

The reviews consistently identified the importance of the multidisciplinary team, whose main members were nurses, physicians, and social workers (details in Online Appendix 5). Fourteen reviews reported on the inclusion of physiotherapists in multidisciplinary teams, underscoring the importance of functionality and rehabilitation as a continuum in EoLC for older people.^87^ Only five reviews mentioned the involvement of volunteers in service delivery, thus limiting the consideration of this resource, particularly for LMICs. Reviews seldom specified the integration of services, especially of health and social care, to ensure continuity of care.

Exceptions were service delivery models that specifically included a social worker (details in Online Appendix 5) and social care models that were embedded in older people's everyday lives, for example, a residential care facility.^70^ Most service models fell on a continuum of service delivery consisting of specialist palliative care, hospital care, long term‐care specialists, and geriatrics. Only a minority of models specifically discussed reaching across the whole continuum of primary and secondary generalist palliative care and community participation to specialist palliative care, for example, Singh and Harding's focus on LMICs.^42^ Seven reviews were classified as encompassing both integrated geriatric care and integrated palliative care (see Table 2), which added to our understanding of the potential of a continuum of palliative geriatric care. These reviews tended to concentrate on social care for older people requiring palliative care that concerned, for example, case and/or care management^43^, ^73^ and social work^67^ or multidisciplinary provision,^54^ or took a broader approach to palliative care encompassing all life‐threatening conditions and models of palliative care.^17^, ^40^ The corresponding outcomes reported included typical geriatric outcomes of function and palliative care outcomes of quality of life,^73^ and/or specific symptoms and concerns,^17^, ^67^ or broad outcomes of health‐related quality of life^40^, ^43^, ^54^ (details in Online Appendix 6).

### Target Outcomes

**Figure 2:**
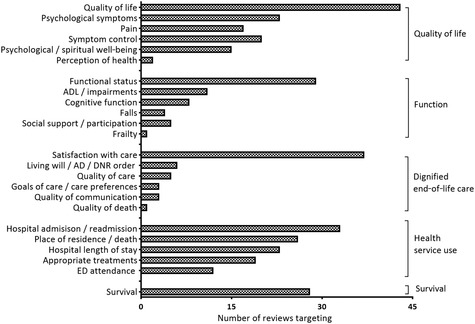
Target Outcomes Ordered by Domain and Frequency of Reporting Abbreviations: ADL, activities of daily living; AD, advanced directive; DNR, do not resuscitate order; ED, emergency department.

In total, 117 separate outcomes were measured across the reviews (details in Online Appendix 6). Only 25 of 117 (21%) were used in a meta‐analysis. Outcomes were grouped into five main themes: quality of life (including symptoms and well‐being), functional outcomes, dignified EoLC, health service use and costs, and survival (Figure 2). The most commonly targeted outcomes were quality of life (43 of 72, 60%), satisfaction with care (37 of 72, 51%), survival (28 of 72, 39%), physical function (25 of 72, 35%), health care costs (25 of 72, 35%), hospital length of stay (23 of 72, 32%), and psychiatric symptoms (23 of 72, 32%). Both Integrated Geriatric Care and Integrated Palliative Care placed equal emphasis on measures for quality of life (17 of 25, 68%, versus 19 of 30, 63%) and health service use (17 of 25, 68%, versus 19 of 30, 63%). The overarching models showed variation in outcome measures relating to symptoms that were more common in Integrated Palliative Care (21 of 30, 70%, versus 11 of 27, 44%) and to physical function and survival, which were more common in Integrated Geriatric Care (15 of 25, 60%, versus 8 of 30, 27%, and 14 of 25, 56%, versus 12 of 30, 40%, respectively). Reviews across the classifications considered survival in order to assess both increasing longevity and as a proxy for the potential harm of shortening life.

### Impact of Service Delivery Models

Table 3 details the results from the meta‐analyses and narrative syntheses for the outcomes and impacts reported. We present these data in relation to the proposed overarching classifications, key processes, and components. Meta‐analyses were more frequently conducted in recent studies (2000‐2010, *n* = 10; 2011‐onward, *n* = 18). There was no discernible trend in reported effectiveness according to the date of publication.

**Table 3:** Reported Impact of Common Service Delivery Models

			Overarching Classifications		Key Processes			Key Components					
Outcome	Narrative Findings	Meta‐analysis	Integrated Geriatric Care	Integrated Palliative Care	Comprehensive Assessment	Case Management	Collaborative Working	Person‐Centered Care	Education	Workforce	Meta‐analysis Findings	Findings and Interpretation	
Quality of life	Quality of life	Lorenz et al. 2008^16^						X	X	X	Pain	Effect size 0.13 (95% CI 0.11 to 0.63)	Effective
	Effective: 17, 40, 41, 42, 46 ^,^ 50, 58, 67, 80 ^,^ 83, 84, 88, 103												
	Inconsistent: 20, 56, 69, 84 ^,^ 89, 98, 99, 101 ^,^ 102, 104, 113												
	No effect: 61, 64, 71, 79												
		Conroy et al. 2011^47^				X					QOL‐ physical	MD 0.2 (95% CI 1.9 to −2.3)	Effective
											QOL‐mental component	MD 0.6 (95% CI 1.3 to −2.5)	Effective
		De Coninck et al. 2017^57^	X				X	X			Fear of falling	SMD 0.17 (95% CI 0.29 to 0.05)	Effective
		Kavalieratos et al. 2016^40^	X	X	X	X	X	X	X		QOL (1‐3 month)	SMD 0.46 (95% CI 0.08 to 0.83)	Effective
											Symptom burden 1to 3 months, 10 studies	SMD −0.66 (95% CI −1.25 to 0.07)	Effective
	Symptoms	Haun et al. 2017^59^	X	X		X	X	X	X	X	QOL ‐health related	SMD 0.27 (95% CI 0.15 to 0.38)	Effective
	Effective: 16, 36, 38, 39, 40 ^,^ 42, 46, 50, 58 ^,^ 61, 67, 70, 72 ^,^ 84, 85, 86, 87, 88, 97										Levels of depressive symptoms	SMD −0.11 (95% CI −0.26 to 0.03)	Effective
	Inconsistent: 16, 17, 41, 44 ^,^ 48, 56, 68, 77 ^,^ 79, 80, 82, 83 ^,^ 89, 92, 93, 103												
	No effect: 14, 71												
											Intensity of symptoms	SMD −0.23 (95% CI −0.35 to −0.10)	Effective
		Phillips et al. 2004^94^	X			X	X	X	X		QOL	P = 0.01 (no other figures given)	Effective
		Ekdahl et al. 2015^55^			X			X	X		Depression	SMD 0.17 (P = 0.02)	Effective
		Fox et al. 2012^53^	X					X			Delirium	RR 0.73 (95% CI 0.61 to 0.88)	Effective
		Higginson et al. 2003^62^		X			X				Pain	OR 0.38 (95% CI 0.23 to 0.64)	Effective
											Other symptoms – effective	OR 0.51 (95% CI 0.30 to 0.88)	Effective
Function	Effective: 67, 70, 83, 84 ^,^ 87, 88, 97, 99	Stuck et al. 2002^51^			X	X					Functional decline	RR 0.76 (95% CI 0.64 to 0.95)	Effective
	Inconsistent: 18, 44, 46, 68 ^,^ 71, 74, 78, 89												
	No effect: 14, 49, 76												
		Conroy et al. 2011^47^				X		X			Function	SMD 0.41 (95% CI 0.21 to 0.61)	Effective
		De Coninck et al. 2017^57^	X				X	X			IADL	SMD 0.30 (95% CI 0.50 to 0.11)	Effective
											Mobility	SMD 0.45 (95% CI 0.78 to 0.12)	Effective
											Disability	SMD 0.19 (95% CI 0.4 to 0.06)	Effective
											Social participation	SMD 0.44 (95% CI 0.69 to 0.19)	Effective
		Ekdahl et al. 2015^55^			X		X	X	X		Personal ADLs	SMD 0.21 (95% CI 0.05 to 0.37)	Inconsistent
		Fox et al. 2012^53^	X					X			Falls	RR 0.51 (95% CI 0.29 to 0.88)	Effective
											Functional decline at discharge	RR 0.87 (95% CI 0.78 to 0.97)	Effective
		Ellis et al. 2011^52^	X			X	X	X	X		Dependence	OR 0.94 (95% CI 0.81 to 1.10)	No effect
											Activities of daily living	SMD 0.06 (95% CI −0.06 to 0.17)	No effect
											Cognitive function	SMD 0.08 (95% CI 0.01 to 0.15)	Effective
											Death or deterioration	OR 0.76 (95% CI 0.64 to 0.90)	Effective
Dignified EOLC	Effective: 14, 17, 36, 40 ^,^ 41, 42, 46, 58 ^,^ 61, 70, 72, 75 ^,^ 76, 80, 81, 83 ^,^ 85, 90, 91, 93 ^,^ 95, 98, 103, 104	Higginson et al. 2003^62^		X			X				Caregiver satisfaction	OR 0.17 (95% CI 0.03 to 0.96)	Effective
	Inconsistent: 18, 48, 50, 56 ^,^ 60, 68, 71, 74 ^,^ 77, 99										Patient satisfaction	Numbers not given	No effect
	No effect: 44, 79, 84, 96												
Health service use	Effective: 14, 17, 44, 50 ^,^ 56, 58, 61, 64 ^,^ 65, 67, 82, 83 ^,^ 85, 87, 93	Stuck et al. 2002^15^				X					Admission to nursing home	High‐intensity care (> 9 visits)	Effective, high intensity
	Inconsistent: 16, 18, 40, 59 ^,^ 69, 71, 73, 74 ^,^ 76, 84, 90, 91 ^,^ 97, 104											RR 0.66 (95% CI 0.48 to 0.92)	No effect, low intensity
	No effect: 79, 95											0‐4 visits RR = 1.05 (95% CI 0.85 to 1.30)	
		Conroy et al. 2011^47^				X		X			Institutionalization	RR 0.75 (95% CI 0.44 to 1.29)	No effect
											Readmission	RR 0.95 (95% CI 0.83 to 1.08)	No effect
		Fox et al. 2012^53^	X					X			Shorter hospital stay	MD 0.61 (95% CI 1.16 to 0.05)	Effective
											Discharge to a nursing home	RR = 0.82 (95% CI 0.68 to 0.99)	Effective
											Discharge to home	RR = 1.05 (95% CI 1.01 to 1.10)	Effective
		Phillips et al. 2004^94^	X			X	X	X	X		Readmission	RR 0.75 (95% CI 0.64 to 0.88)	Effective
											Length of stay	RR −0.37 (95% CI 0.15 to 0.60)	No effect
		McAlister et al. 2004^54^	X	X		X	X	X	X		All‐cause hospitalization	RR 0.84 (95% CI 0.75 to 0.93)	Effective
		Shepperd et al. 2016^60^		X				X	X		Dying at home	RR 1.33 (95% CI 1.14 to 1.55)	Effective
		Gomes et al. 2013^48^		X				X	X		Death at home	(P value = 0.02)	Effective
											Death not in nursing home	(P value = 0.60)	No effect
Survival	Effective: 97	Stuck et al. 2002^15^				X					Mortality: Younger study populations for ages 72.77 to 77.5	RR 0.76 (95% CI 0.65 to 0.88)	Effective
	Inconsistent: 18, 48, 74										Older study populations for ages 80 to 81.6 years	RR 1.09 (95% CI 0.92 to 1.28)	No effect
	No effect: 14, 64, 71, 73, 90												
		Conroy et al. 2011^47^				X		X			Mortality	RR 0.92 (95% CI 0.55 to 1.52)	No effect
		Kavalieratos et al. 2016^40^	X	X	X	X	X	X	X		Survival	HR 0.90 (95% CI 0.69 to 1.17)	No effect
		Phillips et al. 2004^94^	X			X	X	X	X		Mortality	RR 0.87 (95% CI 0.73 to 1.03)	No effect
		Haun et al. 2017^59^	X	X		X	X	X	X	X	Mortality	HR 0.85 (95% CI 0.56 to 1.28)	No effect

Abbreviations: CI, confidence interval; HR, hazard ratio; IADL, instrumental activities of daily living; MD, mean difference; OR, odds ratio; RR, risk ratio; SMD, standardized mean difference; QoL, quality of life.

Forty‐seven reviews analyzed the impact on quality‐of‐life outcomes, nine of which reported meta‐analyses. Pooled analyses were reported for health‐related quality of life (physical and psychological) (*n* = 4/9) and individual and/or multiple symptoms (*n* = 5/9). All pooled estimates of effect demonstrated effectiveness on quality‐of‐life outcomes. Effectiveness on health‐related quality of life was evident for the overarching classifications of Integrated Geriatric Care 40, 53, 57, 59, 94 and Integrated Palliative Care,^40^, ^59^, ^62^ the respective key processes of comprehensive assessment, case management and collaborative working, and key components (see Table 3).

Twenty‐eight reviews reported a narrative synthesis focusing on quality of life (excluding symptoms): 13 reported the models were effective; 11 found inconsistent evidence; and 4 found no effect. Symptoms were reported in narrative form in 37 of the reviews, with 19 finding the model(s) to be effective, 16 finding inconsistent results, and 2 finding no effect.

Impact on function was reported in 25 reviews, with 6 providing a meta‐analysis. Effectiveness on function for the classification of Integrated Geriatric Care was evident in three reviews,^52^, ^53^, ^57^ but two also found inconsistency^55^ and no effect,^52^ dependent on the population, the model's characteristics, and the outcome measured (see Table 3). Nineteen reviews provided consistent narrative findings related to function (see Table 3). The impact on dignified end‐of‐life care was reported by 39 reviews. One pooled quantitative satisfaction ratings and found an effect for caregiver but not patient satisfaction.^62^ Of the remaining 38 reviews that narratively synthesized the impact on dignified EoLC, 24 found an effect; 10 were inconsistent; and 4 reported no effect.

Mortality was assessed in 14 reviews, 5 of which performed meta‐analysis, and all but one found no effect while one found reduced mortality in a subgroup of “younger” older people. Nine other reviews reported on survival narratively. Most reported no effect or inconsistent findings, with only one reporting that the model of care reduced mortality. In no cases were the models found to be associated with increased mortality.

### Scalability and Sustainability

Forty‐four reviews (44 of 72, 61%) provided information to assess the potential scalability and sustainability of the service delivery models. However, only four reviews included studies from LMICs incorporating Zambia,^41^ South Africa,^39^, ^41^ Uganda,^39^ Kenya,^40^ India,^41^, ^42^ Pakistan,^42^ and Nepal.^42^ All reported Integrated Palliative Care, with only two classified also as Integrated Geriatric Care.^40^, ^42^ This limits conclusions about the provision of geriatric service delivery models and how they could be provided. Scalability and sustainability require an understanding of the population, services, and resources available to align models of care with these contextual details.^45^ Important contextual factors for scalability and sustainability were the social and cultural characteristics of the populations studied, the health structural systems, and the definitions of palliative care.^14^, ^36^, ^65^, ^70^, ^77^, ^80^, ^84^, ^90^, ^95^

The reviews offered important insights into key components to support the scalability and sustainability of service delivery models. A public health approach in India became a promising model for increasing access to Integrated Palliative Care through formal health systems, family and community support, and public engagement.^42^ Three reviews focused on sustainability in rural areas and cited the pivotal role of community services to develop palliative care.^41^, ^46^, ^81^ A close working relationship between palliative care and other primary care providers was associated with improved quality of life and more dignified EoLC.^41^, ^46^ The reviews identified approaches to widening the provision of palliative and geriatric care, such as education and training for primary care clinicians and other generalists in the community,^41^, ^46^ and the role of family physicians to develop and implement strategic health programs for aging communities.^81^

## Discussion

Our findings revealed two overarching classifications of integrated care comprising models that focus primarily on either Integrated Geriatric Care or Integrated Palliative Care. We found evidence of effectiveness for both approaches on the main outcome of quality of life, but with different emphases on attainment. The Integrated Geriatric Care model placed greater emphasis on outcomes to improve physical function, while Integrated Palliative Care tended to focus more on outcomes of symptom severity and psychosocial and/or spiritual concerns. But both approaches had areas of synergy in the processes of service delivery, notably person‐centered care and the education of staff and patients and/or caregivers. We identified three overarching processes to integrate care: comprehensive assessment, case management, and collaborative working. Our findings indicate the opportunity of integrating the two approaches with service use tailored to patient needs and intended goals. Integrated care is conceptualized as services, disciplines, commissioners, and policymakers working together to align services and packages of care with older people's needs.^1^ There are exemplars of these types of integrated approaches across the care continuum based on needs and benefits for older people, for example, those with COPD,^106^ heart failure,^107^ dementia,^108^ frailty and multimorbidities,^109^ and a need for long‐term care.^110^, ^111^

Our findings indicate that person‐centered care should drive access to services. The differences in the models’ intended outcomes raises the question of when a person could benefit from each approach. We argue that service use is best triggered by individual need and intention to live life as well as possible by maintaining physical and cognitive function and autonomy (functional and decision making) until the time of death.^112^ This marks a shift away from temporal indicators like prognostication. This integrated approach requires a paradigm shift in older age and EoLC from within (or even from) the alignment with prognostication, to allowing costing and service access based on perceived needs, individual goals, and intended outcomes. It is widely advocated that palliative care be delivered on the basis of need and benefit, not diagnosis or prognosis.^15^, ^112^ This places person‐centered care at the core of care delivery and emphasizes a people‐centered approach to health system design and delivery.^111^, ^113^

### Centrality of Person‐Centered Care

Care that is driven by the person's priorities and goals requires impeccable assessment to encompass the breadth of needs, an understanding of the goals of “what matters to you” (the person), and involving the person in decision making about care and treatment.^112^ Such care enables a pursuit of quality of life by attuning care provision and its goals to the person's own needs and priorities. The use of person‐centered outcome measures in routine care is advocated to assess systematically individual needs, priorities, and goals; to direct the provision of care; and to measure the outcomes of care from the perspectives of the patients and their families, thereby promoting autonomous decision making, quality, and equity.^114^ The likely impact on clinical care, however, requires the use of measures developed and validated for the respective population and setting and the staff's engagement.^115^, ^116^ The Integrated Palliative care Outcome Scale for Dementia (IPOS‐Dem) is an example of a person‐centered measure for palliative geriatric care with clinical validation for use in care homes for people with dementia and multimorbidities.^117^, ^118^ The measure is an outgrowth of the established and validated Palliative care Outcome Scale (POS) family of measures,^119^, ^120^ widely used internationally, including for LMICs in the African and Thai POS, for example.^121^, ^122^

To live life as well as possible in old age and enable healthy dying require the integration and coordination of care between services across the care continuum.^123^ A model of comprehensive palliative care integrated with other specialties like geriatric care is advocated for meeting the multidimensional needs of individuals (and their families) living with multiple long‐term conditions.^112^ With advancing age, a person's needs often increase in both prevalence and severity associated with living with several morbidities and frailty. Consequently, the attainment of these goals often requires more resources and greater effort, by both the patient and his or her caregivers. In turn, this necessitates balancing the management of symptoms and concerns and preserving autonomy, especially by maintaining the person's physical function.^112^

Our analysis conceptualizes Integrated Geriatric Care as person‐centered care with an emphasis on strengthening and maintaining the person's intrinsic capacity and functional ability, and/or reversing the causes of acute decline.^123^ Attention to both maintaining function and managing acute decline is important. Acute decline is common when living with frailty, which is associated with diminishing physiological reserves and increasing risk to a seemingly minor health event like an infection leading to poor outcomes, such as end of life.^3^ However, emphasis on an integrated approach must encompass management of symptoms and concerns and not neglect care of the dying as part of the continuum from healthy aging to healthy dying. The dying phase often is the time of greatest need for the person and his or her family.^112^ Integrated Palliative Care encompasses both specialist and generalist palliative care. Specialist palliative care includes hospice care (with inpatient hospice, day hospice, hospice at home) as well as a range of other specialist advice, support, and care for patients with complex problems.^124^ Generalist palliative care is provided by professionals with basic training in palliative care supporting patients with life‐threatening conditions.^15^

### Effectiveness of Models of Care

Improvement in older people's quality of life is apparent from receipt of both Integrated Geriatric Care^40^, ^57^ and Integrated Palliative Care.^40^ Effectiveness is also evident in the two approaches’ main focus on outcomes to improve function^52^, ^53^, ^57^ and to reduce the severity of symptoms like pain.^59^, ^62^ One systematic review of the Acute Care for Elders model showed effectiveness for both outcomes of function and symptom severity, as well as expenditure data on health service use.^53^ However, all the meta‐analysis data were derived from high‐income countries with no representation of LMICs. Data for LMICs were limited to narrative data from two systematic reviews supporting the effectiveness of Integrated Geriatric Care^42^ and Integrated Palliative Care^41^, ^42^ on quality of life. The effectiveness and sustainability of a service delivery model are critically influenced by how they are implemented in each context, yet these areas are often poorly considered in research evaluating models of care.^125^

The findings reported in the systematic reviews rarely considered the relationship between the outcome of quality of life and how this may be impeded by contextual factors such as poverty. Resource use was rarely collected or reported beyond individuals and health care systems, which are often financed by taxes, social insurance, and private insurance. Informal caregiver costs and the opportunity costs of lost work and lost productivity were not reported. Care and treatment may be initiated by decision making in the clinician‐patient relationship, but its delivery is influenced by the resources available in a society, as well as the configuration and capacity of the delivery system and finance mechanisms,^126^ for example, the availability of national health insurance programs to provide affordable health care.^20^ Personal resources for older people in LMICs are often limited, with the proportion living in poverty above the national average in, for example, 11 out of 15 African countries.^127^ Wider policies and publicly funded safety nets are vital to enable individuals to pursue personal goals and to prevent families from sacrificing basic needs to care for loved ones at the end of life.^113^ Key to maintaining quality of life in old age is the extent to which older people's lives continue to contribute to their families, communities, and environment, however small.^1^ This requires action across sectors to enable individuals to adjust to changes in social positioning, for example, to connect to social networks, maintain function to preserve autonomy, and prevent families from plunging into bankruptcy as they care for their aging relatives.

### Model Delivery

Our findings showed that palliative and geriatric care were delivered through two main overarching processes: collaborative work among settings, services, and disciplines; and case management to coordinate with and tailor care provision to individual needs and priorities. Multidisciplinary teams were common, with three or more disciplines. Multidisciplinary work is vital to maximize function and optimally manage symptoms and concerns. This requires integration across the continuum of EoLC for older people to maintain quality of life and achieve a dignified death. However, the service delivery models minimized consideration of continuity of care across settings such as between secondary and primary care, integration between specialists in palliative and geriatric care, or between specialists and generalists in palliative care.

Transitions between care settings are common for older people nearing the end of life, particularly moving from home to hospital in high‐income countries.^128^, ^129^ The degree of integration among settings, specialties, and services is a key system feature that influences both the quality of care delivered and the outcomes. Poor integration leads to fragmentation of services. In turn, this can impede the coordination of services and late delivery of services, like palliative care, as well as the duplication of services, such as diagnostic tests.^126^ Countries, health care systems, and institutions, therefore, must define “integration” as aligned with the local population's needs and characteristics and primary resources.^15^, ^130^ This definition needs to be guided by a common understanding of what is implied by integration for person‐centered and demand‐led health care.^111^, ^131^ Arriving at a common understanding can contribute to a sharper focus and thus direct attention to the most critical investments and policy reforms.

**Figure 3:**
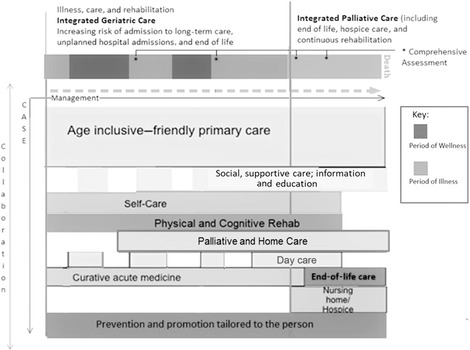
Range of Service Packages for Universal Health Coverage for Older People Nearing the End of Life Modified from a WHO Kobe Center working framework (Ong and Evans 2014^132^).

In an age in which longevity increasingly becomes a norm, health system reforms must focus on accelerating the expansion of health care from primarily the prevention of disease and mortality toward a more comprehensive approach that includes functional ability, quality of life, and a dignified end of life. A diversity of service provision is required to manage effectively the complex and multifaceted needs of older people in order to enable them to live life well and have a healthy death. Figure 3 details the proposed breadth of possible packages of services to deliver the continuum of care across illness trajectories, care settings, and context. These packages attempt to widen access to palliative care for people with chronic progressive conditions across care settings by combining the requirements for UHC to ensure access to affordable palliative care for all, and they realize the World Health Assembly (WHA) Palliative Care Resolution to strengthen palliative care as a comprehensive component of services throughout the life course.^11^

The commonality of education as a key component of service delivery models, reported in 72% of reviews (52 of 72), provides an opportunity to capitalize on the specialties’ reciprocal learning. Palliative care training and education for all staff are essential to realizing the WHA resolution on the integration of palliative care in all health services.^11^ The relief of suffering and the pursuit of an optimal quality of life can and should be offered to older people by all providers of care, including specialties like geriatrics and generalist providers, notably in primary and community care as the main providers of care for older people.^113^ However, most of the evidence underpinning the model in Figure 3 were from high‐income countries with an assumption of available services, for example, primary and community care.

Aging is a relatively new and rapidly increasing epidemiological phenomenon in LMICs, and LMIC health care services are already burdened, for example, with a high prevalence of HIV and the demand for testing and treatment. This places different stressors on health systems and resources compared with those of high‐income countries.^1^ Family caregivers also contend with many competing demands, such as being a subsistence earner and the main carer of orphaned grandchildren.^133^, ^134^, ^135^, ^136^ We must consider the applicability, potential effectiveness, and sustainability of system change and service models across low‐, middle‐ and high‐income settings.^137^ Demographic transitions will change the ways in which we stay healthy, live, and die.^138^ Significant gains in life expectancy mean that more and more older people will live with multiple progressive conditions,^139^ and for many, quality of life may start to matter more than quantity of life.^1^ Accordingly, health systems will be driven to move from a single‐disease framework focusing on cure and prevention toward ensuring equal emphasis on continuity of care across services and specialties and on treatments and care that maintain function, quality of life, and dignity in dying, to enable older people to live meaningful lives despite progressive diseases.^111^, ^139^

### Realigning Health and Social Care Systems

In the 20th century, health care systems developed to improve maternal and child health.^131^ When this was achieved, more middle‐aged persons, having survived childhood, then developed noncommunicable diseases (NCDs).^140^ At this point, health systems altered their focus to tackle early avoidable deaths from NCDs, notably cardiac disease. Many countries, like India, were also newly developing to middle‐income countries. With advances from programs of prevention and cure, most people now die in their late 70s and 80s.^138^ This led to the next wave of NCDs, neurological disorders and dementias, arriving. Health care systems readjusted yet again, moving away from investment in hospitals in favor of community and primary care–based approaches.^141^ Such approaches are one of the principal means of ensuring the continuity and coordination of care required for multimorbid progressive conditions and for the optimal use of resources to meet the growing demand.^113^, ^142^

The integrated approach we have presented in this article is an integral facet of health care systems that all countries will require in order to increase performance and align health and social care with the needs of their aging populations.^1^ The linear health care system's developmental approach of attributing change to specific inputs and outputs (eg, immunization programs) may have been effective in the 20th century, but in the 21st century, a systemwide program is required. Because the global health care system faces many challenges, all health systems, whether in low‐, middle‐, or high‐income countries, must plan their health care systems, allocate their resources, develop health in all sector policies, and train their staff in a different way. It is incumbent on 21st‐century health care systems to advance with people‐centered perspectives in mind and as part of their planning strategies.^111^ The values that underpin people‐centered approaches to health care systems will serve to maximize quality of care. This is applicable for health care systems managing high child‐disease burdens, as well as those in countries with more equal ratios of older people to children,^111^ a key change as societies experience increasing longevity.^138^

Our findings indicate crucial components for models in LMICs, notably the development of community services, particularly in rural areas,^41^, ^46^ as well as health and education policies to integrate palliative and geriatric care across health and social care services by training primary care providers and broadening public engagement.^42^, ^81^ Volunteers play a vital role in community‐oriented programs and public engagement. They act as an indicator of community involvement. Their presence can enable members of the community to engage with palliative and geriatric care services, particularly in communities in which access to services is limited by low levels of health literacy and likely a concurrent lack of understanding of older populations’ needs, especially in late life.^112^, ^143^ Nonetheless, only five systematic reviews reported service delivery models that involved volunteers, and only two included LMICs.^41^, ^42^

The findings for a model of integrated palliative care align with the Lancet Commission's essential package of palliative care to deliver community‐oriented programs for LMICs.^113^ The package is designed to be sustainable by locating changes to health care systems within the wider policy context to address, for example, poverty and by incorporating the lowest cost, such as for medication, and emphasizing practitioners’ competencies rather than their disciplines.^113^ Emphasis is placed on building competencies and including the principles of palliative care in other specialized (eg, geriatrics) and generalists’ services, notably family physicians and community health workers, by providing the necessary training and medical supervision to enable the integration of palliative care across services. There is evidence of the effectiveness of this integrated model in, for example, training generalist nurses to deliver palliative care in HIV care and treatment in Kenya.^144^

It is imperative for LMICs to increase the provision of geriatric care through training of generalists in geriatric care, both in primary care and as part of community‐based services. The reviews that encompassed LMICs focused mainly on the provision of palliative care models of service delivery, with little discussion of the role of geriatric services. Geriatric services are a relatively recent priority in LMICs. For example, Nigeria opened its first dedicated service in 2012, but provision remains sparse to meet the needs of a burgeoning older population of approximately 17 million people.^20^ To achieve the current and projected aging in LMICs and to meet the UHC^145^ and the WHA resolution,^11^ the WHO's public health palliative care strategy (and the putative model of integrated geriatric and palliative care proposed) may need to include the African model of policy and education and the Indian model of community integration. However, this strategy can be achieved only through local research^146^ that takes account of local systems, cultural preferences, and resources.

### Methodological Reflections

Aging, quality of life, and end of life are broad concepts, and we defined and operationalized each in this review. We used the WHO's definitions and international consensus statements to guide our choices and then worked with information specialists to limit the extent to which our choices narrowed our scoping of the evidence. We limited our search strategy from 2000 to assess current service delivery models and the evidence base for them. By drawing on systematic reviews, we determined that the date limitation would not prohibit the inclusion of earlier studies. However, in keeping with our contemporary focus, we found that the majority (49 of 72, 68%) of our included reviews limited their search strategy to 2000 and beyond.

Although our electronic search included publications in all languages, it was limited to three databases that were primarily indexed on English‐language publications. Relying exclusively on database searching is unreliable when trying to identify literature regarding complex interventions,^147^ so our extended search strategy included scanning reference lists, using personal knowledge, and making external contacts. Specifically, we called on experts and active researchers across Africa and Latin America to share gray literature and to scope local databases on our behalf. We also drew on resources to translate non‐English language reviews as required, for example, Ruiz Ingez and colleagues published in Spanish.^93^ The relative gap in knowledge reported from LMICs may reflect the perspective taken by our review teams, as well as the limited dissemination of relevant evaluation studies impeded by the resources and expertise required to undertake effectiveness evaluations. Our main findings on the overarching processes to enhance outcomes of care, however, were corroborated by an international review of integrated health services promoting a people‐centered approach.^111^

Our choice to scope systematic reviews allowed us to incorporate multiple search strategies, including our own search strategies and those from the 72 systematic reviews we included. We are therefore confident that this represents a comprehensive review of the published literature on the effectiveness of service delivery models to maximize the quality of life for older people in their last 1 or 2 years of life. There was potential for primary studies to be included in more than one review, and we did not explicitly check for duplication among the reviews, which may have caused us to overestimate their effectiveness. Drawing on systematic reviews rather than primary studies also limited our ability to describe the granularity of the model components and processes of delivery, for example, referral criteria or triggers, and the relationships between outcomes such as physical and mental suffering. Often the reviews reported on different service delivery models grouped by their setting or overarching aim, thus requiring us to interpret model components from the narrative descriptions in the text. The case examples we have provided do offer more information and help service providers understand how care might be integrated. In addition, we demonstrated the potential effectiveness of our proposed classification on quality of life and the key processes and components. We could not, however, confidently compare model components or directly link different models to the reported outcomes. These are important future areas of research to inform policy and practice and to examine our proposed conceptual model of integrated palliative care and integrated geriatric care.

## Conclusion and Implications

We identified two overarching classifications of integrated geriatric and palliative care to maximize older people's quality of life at the end of life. Both models had similar orientations to improving quality of life, but with differing emphases on the outcomes of either physical and mental functions and symptoms and psychosocial and/or spiritual concerns. Essential to both models was care centered on the person and the education of staff, patients, and caregivers.

This work has several implications. Our findings highlight the urgent need for health system change to improve EoLC as part of UHC. This is an imperative to improve the continuum between healthy aging and healthy dying, which is a challenge that all health care systems face, either now or in the next generation globally as societies age. Achieving UHC coverage is the most important means to advance health and well‐being and align services with the global aging population's needs and priorities. Our findings underscore the necessity of integrated palliative and geriatric services, to optimize the function and management of symptoms and concerns by working together in a coordinated and collaborative way and seeking reciprocal learning to connect the continuum between healthy aging and healthy dying. Emphasis on integration must not, however, neglect care of the dying, which is often a time of the greatest need. Integrating services requires a system change driven by a person‐centered approach that takes account of individual circumstances, values, and priorities rather than diagnosis or prognosis. This is crucial at a time of increasing longevity, when providing a continuum of services to support individual goals becomes more important than curing disease or saving life. Access to palliative care (and the application of its principles of care to geriatric services) is needed before the last months of life, driven by goals of care and individual priorities. An increase in geriatric care provision for older people is required to maintain and optimize function as a continuum throughout late life until death.

Countries should validate the key resources of person‐centered measures for palliative geriatric care and implement them in routine care to help care providers assess their patients’ needs and likelihood of benefits and to inform quality indicators and outcome measures. In clinical practice, evidence supports service providers maintaining a primary focus on quality of life to enable individuals to do things they value and continue to find meaning in their lives across the life course, especially in later life when quality of life may matter more than mere quantity. We recommend that providers of services for this population routinely measure experiences of care and involvement in shared decision making, to explore the extent that individual priorities are considered and drive care provision, and to measure key outcomes of, for example, quality of life. Future research is required to review primary studies and link different service delivery models to different health outcomes and to test and identify the key components of integrated person‐centered palliative geriatric care to form a theoretical model. A discrete choice experiment would improve our understanding of the priorities of older people at the end of life, what they value more and value less, and how they would like future services to be delivered. We found little evidence for resource‐limited settings in LMICs, even though these areas have the most rapidly aging populations and the greatest needs. We recommend primary studies evaluating models of care in LMICs, prioritizing trials of clinical and cost‐effectiveness in order to provide evidence to guide the development of appropriate models of “integration” of services that are aligned with disease epidemiology, available resources, and sociocultural practices. Because cost is pivotal to sustainability, we encourage economic analyses that span health and social care and include all sources of finance to understand inequalities in accordance with the local context.

Finally, by tackling these issues of continuity and coordination of care for older people, countries are protecting their health care systems against future needs, as well as improving their current systems and approaches. The principles of people‐centered integrated care are relevant where maternal/child health issues predominate and become imperative when longevity is achieved. This is precisely why this continuum for the coordination of care between geriatric and palliative care is an ideal platform for studying the health systems of the 21st century that must deliver integrated person‐centered services.

## Abbreviations

ADLs: Activities of Daily Living
A&E: Accident and Emergency
AMSTAR: A Measurement Tool to Assess Systematic Reviews
CATWOE: Customers, Actors, Transformation World View, Owner, External influences
COPD–WHO: Chronic Obstructive Pulmonary Disease–World Health Organization
EoLC: End‐of‐Life Care
IADL: Instrumental Activities of Daily Living
ICU: Intensive Care Unit
LMICs: Low‐ and Middle‐Income Countries
MeSH: Medical Subject Headings
PRISMA: Preferred Reporting Items for Systematic Reviews and Meta‐Analysis
QoL: Quality of Life
UHC: Universal Health Coverage
WHA: World Health Assembly

## Supporting information


**Appendix 1**. Search Strategy for Medline
**Appendix 2**. Author Year and Aim by Included Systematic Review
**Appendix 3**. Countries Included in Systematic Reviews by WHO Region
**Appendix 4**. AMSTAR Quality Appraisal by Included Systematic Review
**Appendix 5**. Service Model Providers by Respective Systematic Review
**Appendix 6**. Target Outcomes Reported in the Systematic Reviews
