# Supplementary material for: Service Delivery Models to Maximize Quality of Life for Older People at the End of Life: A Rapid Review
Source: Milbank Q. 2019 Mar 18;97(1):113–75. doi: 10.1111/1468-0009.12373 (PMC6422603; doi:10.1111/1468-0009.12373)
Supplement: Supplementary file 1 — Appendix 1. Search Strategy for Medline Appendix 2. Author Year and Aim by Included Systematic Review Appendix 3. Countries Included in Systematic Reviews by WHO Region Appendix 4. AMSTAR Quality Appraisal by Included Systematic Review Appendix 5. Service Model Providers by Respective Systematic Review Appendix 6. Target Outcomes Reported in the Systematic Reviews [file MILQ-97-113-s001.docx]

**Appendix 1**

**Search Strategy for Medline**

|  | **Population End of Life/ Advanced Disease** | **Intervention (eg, Hospital)** | | **Outcome** |
| --- | --- | --- | --- | --- |
| MESH terms | Exp Terminally ill/  Exp Terminal care/  Palliative Care/  Frailty | Exp Patient admission/  Exp Patient readmission/  Geriatric nursing/  Primary nursing/  Hospice and palliative care nursing/  Exp nursing services/  Symptom assessment/  Geriatric assessment/  Needs assessment/  Hospital volunteers/  Nursing process/  Exp patient care planning/  Exp Progressive patient care/  Exp Caregivers/  Exp Home care services/  Exp Hospice care/  Exp Patient care team  Exp Continuity of patient care/ | | Exp Quality of life/  Exp Pain/  Exp Pain management/  Exp Dyspnea/  Exp Anxiety/  Exp Anxiety disorders/  Depression/  Exp Depressive disorder/  Personal satisfaction/  Exp Activities of daily living/  Constipation/  Accidental Falls/  Exp Mental health/  Exp Social isolation/  Exp Social support/  Exp Patient satisfaction/  Exp Budgets/  Exp Costs and cost analysis/  Economics/  Exp Economics hospital/  Exp Economics medical/  Economics nursing/  Exp Fees and charges/  Exp Resource allocation/  Value of life/ |
| Key terms | EoL.tw  End?of?life.tw  Dying.tw  Palliative.tw  Last adj4life.tw  Hospice.tw  Life limit*tw  Advanced disease*tw  Palliative treatment.tw  Palliative medicine.tw  Terminal care.tw  Terminally ill.tw  End-of-life care.tw  Hospice care.tw  Palliation.tw.  Palliative care$.tw. Multi*morbidity.tw  Co*morbidity.tw  ((Frail old*) AND (people OR adult* OR person*)).ti,ab  Frail*.tw  Frail elder*.ti,ab  Frailty syndrome*.ti,ab  Advanced illness.tw | Integrated care.tw  Model adj4care.tw  Multi?disciplin*tw  Multi?disciplinary team.tw  Volunteer* tw  Volunt*tw  Hospital adj3home.tw  Comprehensive assess*tw  Holistic assess*  (special$ adj2palliat$).tw.  Nurse-led.tw  Co?ordination adj3care.tw  Care plans.tw  Care?giver*.tw  Person?centr*.tw  Self?manage*.tw  Community health worker*.tw  Service delivery.tw  Community?based.tw  Home visit*.tw  Case management.tw  Care management.tw | | Good death.tw  Symptom*.tw  Concern*.tw  Attainment  Dignity.tw  Empowerment.tw  Transition*.tw  Pain.tw  Dyspn?ea.tw  Breathless*.tw  Anxiety.tw  Anxious.tw  Depress*.tw  Quality of life.tw  Qol.tw  (quality adj2life).tw  Distress.tw  Wellbeing.tw  ADL*.tw  Activities of daily living.tw  Constipat*.tw  Fall*.tw  Mobil*.tw  Symptom management.tw  Psychosocial.tw  (psycho adj social).tw  Psychological distress.tw  Enablement.tw  Mastery.tw  Resilience.tw  Stress.tw  Financ*tw  (Cost* or economic*).ti  (Cost* adj2(effective* or utilit* or benefit* or minimi*)).ab  Economic model*.tw  (Budget* or fee* or financ* or pricing or price* or resource* allocat* or (value adj2(monetary or money))).ti,ab |
| Boolean operators | OR | OR | | OR |
|  | AND | | AND | |
| Limit | ((Overview*.ti OR Review.ti OR Synthesis.ti OR Summary.ti OR Cochrane.ti OR Analysis.ti) AND (reviews.ti OR meta-analyses.ti OR articles.ti OR umbrella.ti)) OR ,,umbrella review,,.ti,ab OR (meta-review.ti.ab ORMetareview.ti,ab) OR ((overview*.ti OR Reviews.ti) AND (systematic.ti OR Cochrane.ti)) OR (reviews.ti,ab and (meta.ti,ab OR Published.ti,ab OR Quality.ti,ab OR Included.ti,ab OR summar*.ti,ab)) OR (,,cochrane reviews,,.ti,ab) OR (evidence.ti AND (reviews.ti OR meta-analyses.ti)) | | | |

**Appendix 2**

**Author Year and Aim by Included Systematic Review**

| **Author Year Citation** | **Systematic Review Aim** |
| --- | --- |
|  |  |
| Alcide and Potocky 2015^92^ | To assess the literature on outcomes of hospice social work intervention. |
| Bai et al. 2013^80^ | To identify clinical interventions for improving the quality of life of people with advanced hepatocellular carcinoma. |
| Bainbridge et al. 2016^46^ | To determine which components of in-home end-of-life care programs are most commonly associated with better outcomes than those of usual care. |
| Bakitas et al. 2015^41^ | To synthesize evidence relating to palliative care in rural areas. |
| Bakker et al. 2011^97^ | To review the evidence for hospitalwide interventions for older patients. |
| Brereton et al. 2017^36^ | To identify the existing range of models of palliative care that have been evaluated. |
| Candy et al. 2011^148^ | To identify the current evidence for the effectiveness of hospices and hospice care in a patient’s home and in nursing homes. |
| Candy et al. 2012^91^ | To describe the spiritual and religious interventions for adults in the terminal phase of their disease and to evaluate these effects on their well-being. |
| Carpenter 2017^90^ | To review the care of patients discharged to nursing facilities following hospital-based palliative care consultation. |
| Catania et al. 2015^88^ | To determine to what extent interventions focused on measuring the quality of life in palliative care practice are effective in improving patient outcomes. |
| Conroy et al. 2011^47^ | To assess the role of comprehensive geriatric assessment in older patients who were treated and discharged from hospital. |
| De Coninck et al. 2017^57^ | To assess the effectiveness of occupational therapy to improve performance in daily-living activities in community-dwelling, physically frail older people. |
| Diop et al. 2017^50^ | To characterize the interventions and effectiveness of palliative care for advanced heart failure patients. |
| Dy et al. 2008^98^ | To better understand the conceptualization of satisfaction with end-of-life care and the effectiveness of palliative care interventions for this outcome. |
| Dy et al. 2013^75^ | To investigate whether interventions focusing on continuity coordination and transitions of care for patients with advanced and serious illness improve patient- and caregiver-centered outcomes. |
| Easton et al. 2016^70^ | To identify the structures and processes in residential aged-care settings and, if the costs and outcomes for residents with dementia have been assessed, to determine whether they are cost-effective. |
| Ekdahl et al. 2015^55^ | To summarize the evidence for the effects of comprehensive geriatric assessment initiated in hospital compared with the usual care in a population of frail and moderately frail patients older than 65 who were admitted to hospital for acute care. |
| Eklund et al. 2009^84^ | To review integrated and coordinated interventions targeting frail elderly people living in the community. |
| El-Jawahri et al. 2011^17^ | To review palliative care interventions and their effect on patients, quality of life symptoms, and other end-of-life outcomes. |
| Ellis et al. 2011^52^ | To determine the effectiveness of inpatient comprehensive geriatric assessment for frail older adults admitted to hospital for an unplanned emergency. |
| Fox et al. 2012^53^ | To determine the effectiveness of acute geriatric-unit care based on all or part of the Acute Care for Elders (ACE) model and introduced in the acute phase of illness or injury. |
| Frank and Wilson 2015^81^ | To discuss models of care for frail seniors provided in primary-care settings. |
| Garcia-Perez et al. 2009^85^ | To compare specialized palliative-care models, effectiveness, and cost-effectiveness. |
| Gomes et al. 2013^48^ | To quantify the effect of home palliative-care services on patients’ chances of dying at home. |
| Hall et al. 2011^14^ | To determine the effectiveness of multicomponent palliative-care service delivery interventions for residents of retirement homes. |
| Haun et al. 2017^59^ | To compare the effects of early palliative interventions versus treatment as usual or standard care on health-related quality-of-life symptoms and survival among adults with advanced cancer. |
| Health Quality Ontario 2014^58^ | To determine whether an optimal team-based model of care exists for service delivery at the end of life. |
| Higginson and Evans 2010^61^ | To address whether palliative-care teams improve symptoms and quality of life for both patients with advanced cancer and their caregivers. |
| Higginson et al. 2002^38^ | To determine whether hospital-based palliative-care teams improve the process or outcomes of care for patients at the end of life and their families. |
| Higginson et al. 2003^62^ | To determine the effect of palliative- and hospice-care teams. |
| Hodgkinson et al. 2011^99^ | To identify which staffing models in the long-term aged-care sector are best for patient and staff outcomes. |
| Hopman et al. 2016^71^ | To describe comprehensivecare programs targeting multimorbid and/or frail patients and to estimate their effectiveness in improving patient outcomes, health-care utilization, and costs. |
| Joseph et al. 2016^100^ | To synthesize the evidence for the effectiveness of structured interdisciplinary collaboration on patient satisfaction and hospital admission and readmission rates for adults receiving home-hospice services. |
| Kane et al. 2015^83^ | To examine the evidence for patient-centered care for chronic heart failure. |
| Kavalieratos et al. 2016^40^ | To determine the association of palliative care with quality-of-life symptom-burden survival and other outcomes for both people with life-limiting illnesses and their caregivers. |
| Kim and Tarn 2016^65^ | To investigate the relationship between the involvement of primary care in end-of-life care and health and utilization outcomes. |
| Latour et al. 2007^76^ | To summarize the evidence for the effectiveness of postdischarge, nurse-led case management of complex-care patients. |
| Lorenz et al. 2008^16^ | To assess the evidence for interventions to improve palliative- and end-of-life care. |
| Lowthian et al. 2015^49^ | To examine the effectiveness of emergency-department community-transition strategies to support transition to a safe community. |
| Luckett et al. 2014^63^ | To identify and synthesize international policy recommendations for population-based palliative care and the evidence for improvements for patient, family, and health-system outcomes. |
| Lupari et al. 2011^101^ | To review the research on and the service evaluation of evidence of nurse-led case-management services for older people with multiple chronic conditions, in their own homes. |
| Maharaj and Harding 2016^102^ | To identify and appraise the evidence needs models of care interventions and outcomes of palliative care in the Caribbean. |
| Martinez et al. 2014^86^ | To evaluate the effectiveness of health-care interventions targeting pain in patients with advanced cancer. |
| May et al. 2014^66^ | To review the economic evidence on specialist palliative-care consultation teams in hospitals. |
| McAlister et al. 2004^54^ | To investigate the evidence for the effectiveness of multidisciplinary teams treating heart failure. |
| Nevis 2014^79^ | To review the effectiveness of educational interventions for health-care providers patients nearing the end of life and for informal caregivers to improve patient and informal-caregiver outcomes. |
| Nordly et al. 2016^89^ | To overview the organization and outcomes of home-based, specialized palliative-care for patients with advanced cancer. |
| Oeseburg et al. 2009^69^ | To evaluate the effects of patient case-advocacy case-management on service use and health-care costs for impaired older adults or adults with a chronic somatic disease living in the community. |
| Pham and Krahn 2014^82^ | To evaluate the cost-effectiveness of end-of-life care interventions. |
| Phillips et al. 2013^56^ | To determine the efficacy of comprehensive discharge planning plus postdischarge support for older inpatients with chronic heart failure. |
| Phillips et al. 2004^94^ | To determine the evidence for case conferencing as an intervention to improve palliative-care outcomes for older people living with advanced dementia in nursing homes. |
| Pillotto et al. 2017^18^ | To consider implementing comprehensive geriatric-assessment programs in different health-care settings. |
| *Procter 2012^96^ | To consider how specialist palliative-care professionals can engage with other health professionals to ensure a collaborative approach to end-of-life care. |
| Puts et al. 2017^78^ | To consider the evidence for interventions to prevent and reduce frailty in community-dwelling older adults. |
| Richards and Coast 2003^74^ | To determine the effectiveness and cost of interventions intended to improve access to health and social care for older patients following their discharge from acute-care hospitals. |
| Rizzo and Rowe 2016^67^ | To assess the current evidence for the efficacy and cost-effectiveness of social work interventions in aging. |
| Robinson et al. 2009^39^ | To identify, evaluate, and synthesize the published literature on rural palliative care. |
| Roczen et al. 2016^72^ | To examine whether and how the integration of palliative-care practices into intensive-care units is associated with clinical and nonclinical outcomes. |
| Ruiz-Iniguez et al. 2017^93^ | To assess the effectiveness of nursing interventions for the quality of life of palliative-care patients receiving home-based care. |
| Ryburn et al. 2009^87^ | To review the “real world” potential (ie, efficacy and effectiveness) of restorative approaches to home care for frail older adults. |
| Sampson et al. 2005^95^ | To review the scientific literature on the efficacy of a palliative-care model for patients with dementia. |
| Shepperd et al. 2016^60^ | To determine whether providing home-based end-of-life care reduces the likelihood of dying in hospital and what effect this would have on patients, symptoms, and quality of life. |
| Simoens et al. 2010^45^ | To review literature on the costs of treating terminal patients, with the focus on the level, distribution, and drivers of costs of treating terminal patients |
| Singer et al. 2016^103^ | To explore how payers and providers should identify patients with an “advanced illness” and the specific interventions they should implement. |
| Singh and Harding 2015^42^ | To appraise the evidence for palliative-care models’ interventions and outcomes in South Asia. |
| Soares et al. 2012^104^ | To review the effects of discharge interventions on patients and safety (eg, adverse events) and to evaluate the effects on the effectiveness and efficiency of care processes. |
| Stuck et al. 2002^51^ | To evaluate the effect of preventive home visits on functional-status nursing-home admission and mortality. |
| Windham et al. 2003^73^ | To identify the effectiveness of care-management strategies and outcome measures likely to be helpful in establishing the overall clinical efficacy of congestive heart–failure patients. |
| You et al. 2012^44^ | To evaluate the effects of case management on community aged-care on clients, caregivers, and outcomes. |
| You et al. 2013^43^ | To evaluate the effects of case management in community aged-care interventions on service use and costs. |
| Young et al. 2017^68^ | To assess the effects of long-term home or foster care versus institutional care for functionally dependent older adults. |
| Zimmermann et al. 2008^77^ | To systematically review the evidence for the effectiveness of specialized palliative care. |

**Appendix 3**

**Countries Included in Systematic Reviews by WHO Region**

| **Country** | **Review(s)** | **Total** |
| --- | --- | --- |
| **Region of the Americas** | | |
| USA | ^92,80,46,41,97,64,91,90,88,57,50,70,55,84,17,52,53,85,48,14,59,58,61,62,71,83,40,65,49,101,86,66,54,79,69,94,56,78,74,67,72,93,87,95,60,45,51,44,43,68,77^ | 51 |
| Canada | ^80,41,97,57,70,55,84,17,52,81,48,59,58,61,38,62,99,71,40,65,49,86,54,69,94,74,93,51,44,68,77^ | 31 |
| Peru | ^53^ | 1 |
| Argentina | ^38^ | 1 |
| Jamaica | ^102^ | 1 |
| Barbados | ^102^ | 1 |
| Cuba | ^102^ | 1 |
| Trinidad and Tobago | ^102^ | 1 |
| St Lucia | ^102^ | 1 |
| Grenada | ^102^ | 1 |
| Antigua | ^102^ | 1 |
| Barbuda | ^102^ | 1 |
| Montserrat | ^102^ | 1 |
| St Kitts and Nevis | ^102^ | 1 |
| St Vincent and the Grenadines | ^102^ | 1 |
| Anguilla | ^102^ | 1 |
| Dominica | ^102^ | 1 |
| Dominican Republic | ^102^ | 1 |
| **Europe** | | |
| UK | ^64,90,88,70,17,85,48,58,61,38,62,40,49,101,54,94,96,39,87,60,45,51,44,43,68,77^ | 26 |
| Sweden | ^41,97,55,52,53,48,61,38,62,83,40,54,89,94,56,78,93,68^ | 18 |
| Italy | ^64,84,48,59,61,38,61,65,86,89,69,74,45,44,43^ | 15 |
| Norway | ^80,90,55,17,52,48,58,61,40,60,77^ | 11 |
| The Netherlands | ^88,70,55,99,71,65,86,54,94,51^ | 10 |
| Germany | ^90,88,70,55,52,62,74,39,93^ | 9 |
| Spain | ^53,48,79,78,39^ | 5 |
| France | ^53,38,54,72,45^ | 5 |
| Finland | ^69,44,43^ | 3 |
| Ireland | ^54,89,94^ | 3 |
| Denmark | ^65,51^ | 2 |
| Poland | ^89,93^ | 2 |
| Switzerland | ^51^ | 1 |
| Malta | ^89^ | 1 |
| **Western Pacific** | | |
|  |  |  |
| Australia | ^80,46,41,90,57,70,52,53,85,48,59,58,62,71,40,49,86,54,89,94,56,78,74,39,93,87,45,51,61^ | 29 |
| Japan | ^80,46,64,71,65,78,93^ | 7 |
| Hong Kong | ^80,40,49,45,43,44^ | 6 |
| Tawain | ^78,68^ | 2 |
| China | ^80,43^ | 2 |
| New Zealand | ^54,88^ | 2 |
| Singapore | ^49,78^ | 2 |
| **Eastern Mediterranean** | | |
| Israel | ^46,44,43,45,64^ | 5 |
| **Africa** | | |
| Zambia | ^41^ | 1 |
| South Africa | ^39,41^ | 2 |
| Uganda | ^39^ | 1 |
| Kenya | ^40^ | 1 |
| **Southeast Asia** | | |
| India | ^41,42^ | 2 |
| Nepal | ^42^ | 1 |
| Pakistan | ^42^ | 1 |

**Appendix 4**

**AMSTAR Quality Appraisal by Included Systematic Review**

| **Author Year Citation** | **“A Priori” Design Provided** | **Duplicate Study Selection and Data Extraction** | **Comprehensive Literature Search Performed** | **Status of Publication (ie, Gray Literature) Used as an Inclusion Criterion?** | **List of Studies (Included and Excluded) Provided** | **Characteristics of the Included Studies Provided** | **Scientific Quality of the Included Studies Assessed and Documented** | **Scientific Quality of the Included Studies Used Appropriately in Formulating Conclusions** | **Methods Used to Combine the Findings of the Appropriate Studies** | **Likelihood of Publication Bias Assessed** | **Conflict of Interest Included** | **Total** |
| --- | --- | --- | --- | --- | --- | --- | --- | --- | --- | --- | --- | --- |
| Alcide et al. 2015^92^ | Yes | NR | Yes | Yes | Yes | Yes | Yes | NR | NA | No | No | 6 |
| Bai et al. 2013^80^ | No | NR | No | NR | No | Yes | Yes | Yes | Yes | No | No | 4 |
| Bainbridge et al. 2016^46^ | Yes | No | No | No | No | No | Yes | Yes | Yes | Yes | Yes | 6 |
| Bakitas et al. 2015^41^ | No | NR | No | No | No | Yes | No | Yes | NA | Yes | No | 3 |
| Bakker et al. 2011^97^ | Yes | Yes | No | NR | No | Yes | Yes | Yes | NA | Yes | No | 6 |
| Brereton et al. 2017^36^ | Yes | Yes | Yes | Yes | No | Yes | Yes | Yes | NA | Yes | Yes | 9 |
| Candy et al. 2011^64^ | Yes | Yes | No | No | No | Yes | Yes | Yes | Yes | No | Yes | 7 |
| Candy et al. 2012^91^ | Yes | Yes | Yes | Yes | No | Yes | Yes | Yes | Yes | No | No | 8 |
| Carpenter 2017^90^ | Yes | No | Yes | No | No | No | No | No | Yes | No | No | 3 |
| Catania et al. 2015^88^ | Yes | Yes | Yes | No | No | Yes | Yes | Yes | Yes | Yes | No | 8 |
| Conroy et al. 2011^47^ | Yes | Yes | Yes | No | No | Yes | Yes | Yes | Yes | Yes | No | 8 |
| De Coninck et al. 2017^57^ | Yes | No | Yes | No | Yes | Yes | Yes | Yes | Yes | No | No | 7 |
| Diop et al. 2017^50^ | Yes | Yes | Yes | Yes | No | Yes | No | Yes | Yes | No | Yes | 8 |
| Dy et al. 2008^98^ | No | No | No | No | No | No | No | No | NR | No | No | 0 |
| Dy et al. 2013^75^ | No | Yes | Yes | NR | No | Yes | Yes | Yes | Yes | No | No | 6 |
| Easton et al. 2016^70^ | No | Yes | Yes | Yes | No | No | Yes | Yes | Yes | No | No | 6 |
| Ekdahl et al. 2015^55^ | No | Yes | Yes | No | No | Yes | Yes | Yes | Yes | No | No | 6 |
| Eklund et al. 2009^84^ | No | Yes | No | No | No | Yes | Yes | No | Yes | No | No | 4 |
| El-Jawahri et al. 2011^17^ | No | NR | No | No | No | No | Yes | Yes | Yes | No | No | 3 |
| Ellis et al. 2011^52^ | Yes | Yes | Yes | No | Yes | Yes | Yes | Yes | Yes | No | No | 8 |
| Fox et al. 2012^53^ | Yes | Yes | Yes | No | Yes | Yes | Yes | Yes | Yes | Yes | Yes | 9 |
| Frank and Wilson 2015^81^ | No | NA | NA | NA | NA | NA | No | NA | NR | NA | No | 0 |
| Garcia-Perez et al. 2009^85^ | NR | Yes | Yes | No | No | Yes | Yes | Yes | Yes | Yes | No | 7 |
| Gomes et al. 2013^48^ | Yes | Yes | Yes | Yes | No | Yes | Yes | Yes | Yes | Yes | Yes | 10 |
| Hall et al. 2011^14^ | Yes | Yes | Yes | Yes | Yes | Yes | Yes | Yes | Yes | Yes | Yes | 11 |
| Haun et al. 2017^59^ | Yes | Yes | Yes | Yes | Yes | Yes | Yes | Yes | Yes | Yes | No | 9 |
| Health QualityOntario 2014^58^ | Yes | No | Yes | Yes | No | Yes | Yes | Yes | Yes | No | No | 7 |
| Higginson and Evans 2010^61^ | Yes | No | Yes | No | No | Yes | Yes | Yes | Yes | No | No | 6 |
| Higginson et al. 2002^38^ | Yes | Yes | Yes | Yes | No | Yes | Yes | Yes | Yes | No | No | 8 |
| Higginson et al. 2003^62^ | Yes | Yes | Yes | Yes | No | Yes | Yes | Yes | Yes | Yes | No | 9 |
| Hodgkinson et al. 2011^99^ | Yes | Yes | Yes | Yes | Yes | Yes | Yes | Yes | Yes | Yes | No | 10 |
| Hopman et al. 2016^71^ | Yes | Yes | No | No | No | No | Yes | Yes | Yes | Yes | No | 6 |
| Joseph et al. 2016^100^ | No | NR | No | Yes | Yes | NA | NA | NA | NA | No | Yes | 3 |
| Kane et al. 2015^83^ | Yes | No | Yes | No | No | No | Yes | Yes | Yes | Yes | No | 6 |
| Kavalieratos et al. 2016^40^ | Yes | Yes | Yes | No | No | Yes | Yes | Yes | Yes | Yes | No | 8 |
| Kim and Tarn 2016^65^ | Yes | Yes | No | No | No | Yes | No | No | Yes | No | Yes | 5 |
| Latour et al. 2007^76^ | Yes | Yes | Yes | No | No | Yes | Yes | Yes | Yes | No | No | 7 |
| Lorenz et al. 2008^16^ | No | No | No | No | No | Yes | No | NR | Yes | No | Yes | 3 |
| Lowthian et al. 2015^49^ | Yes | Yes | Yes | No | No | Yes | Yes | Yes | Yes | No | Yes | 8 |
| Luckett et al. 2014^63^ | No | No | No | Yes | No | Yes | Yes | NR | Yes | No | Yes | 5 |
| Lupari et al. 2011^101^ | No | NR | Yes | Yes | No | Yes | No | NR | Yes | No | Yes | 5 |
| Maharaj and Harding 2016^102^ | Yes | No | Yes | No | No | No | Yes | Yes | Yes | Yes | No | 6 |
| Martinez et al. 2014^86^ | No | Yes | No | No | No | Yes | Yes | NR | Yes | No | Yes | 5 |
| May et al. 2014^66^ | No | No | Yes | No | No | Yes | No | NR | Yes | No | Yes | 4 |
| McAlister et al. 2004^54^ | Yes | Yes | Yes | No | No | No | No | No | Yes | No | No | 4 |
| Nevis 2014^79^ | No | No | Yes | No | No | Yes | Yes | Yes | Yes | Yes | Yes | 7 |
| Nordly et al. 2016^89^ | Yes | NR | Yes | No | No | Yes | No | Yes | Yes | No | No | 5 |
| Oeseburg et al. 2009^69^ | Yes | Yes | Yes | No | No | Yes | Yes | Yes | Yes | No | No | 7 |
| Pham and Krahn 2014^82^ | Yes | No | Yes | No | No | No | No | No | Yes | No | No | 3 |
| Phillips et al. 2004^94^ | Yes | Yes | Yes | No | No | Yes | Yes | Yes | Yes | Yes | No | 8 |
| Phillips et al. 2013^56^ | Yes | No | Yes | Yes | No | Yes | Yes | Yes | Yes | No | No | 7 |
| Pillotto et al. 2017^18^ | Yes | No | Yes | Yes | No | No | No | No | Yes | No | No | 4 |
| Procter 2012^96^ | Yes | No | Yes | No | No | No | Yes | Yes | Yes | No | No | 5 |
| Puts et al. 2017^78^ | Yes | Yes | Yes | Yes | No | Yes | Yes | Yes | Yes | No | No | 8 |
| Richards and Coast 2003^74^ | Yes | NR | Yes | No | No | No | Yes | Yes | No | No | No | 4 |
| Rizzo and Rowe 2016^67^ | Yes | NR | Yes | No | No | No | No | No | Yes | No | No | 3 |
| Robinson et al. 2009^39^ | Yes | No | Yes | No | No | No | Yes | No | Yes | No | Yes | 5 |
| Roczen et al. 2016^72^ | Yes | No | No | No | No | Yes | No | No | Yes | No | Yes | 4 |
| Ruiz-iniguez et al. 2017^93^ | No | Yes | Yes | Yes | No | Yes | Yes | No | NA | No | Yes | 6 |
| Ryburn et al. 2009^87^ | Yes | No | No | No | No | Yes | No | NA | Yes | No | No | 4 |
| Sampson et al. 2005^95^ | Yes | No | Yes | Yes | Yes | Yes | No | NA | Yes | No | No | 6 |
| Shepperd et al. 2016^60^ | Yes | Yes | Yes | Yes | Yes | Yes | Yes | Yes | Yes | No | No | 9 |
| Simoens et al. 2010^45^ | Yes | No | No | No | No | Yes | Yes | Yes | Yes | No | Yes | 6 |
| Singer et al. 2016^103^ | Yes | No | No | No | No | Yes | No | No | Yes | No | No | 3 |
| Singh and Harding 2015^42^ | Yes | No | Yes | No | No | No | No | Yes | Yes | Yes | No | 5 |
| Soares et al. 2012^104^ | Yes | No | No | No | No | Yes | No | No | Yes | No | No | 3 |
| Stuck et al. 2002^51^ | Yes | Yes | Yes | No | No | No | Yes | Yes | Yes | No | No | 6 |
| Windham et al. 2003^73^ | No | NR | No | NR | No | No | No | No | Yes | No | No | 1 |
| You et al. 2012^44^ | Yes | No | Yes | No | No | Yes | Yes | Yes | Yes | No | No | 6 |
| You et al. 2013^43^ | Yes | No | Yes | No | No | No | Yes | Yes | Yes | No | No | 5 |
| Young et al. 2017^68^ | Yes | Yes | Yes | Yes | Yes | Yes | Yes | Yes | Yes | Yes | No | 10 |
| Zimmermann et al. 2008^77^ | Yes | Yes | Yes | No | Yes | Yes | Yes | Yes | Yes | No | Yes | 9 |

Abbreviations: NR, not recorded; NA, not applicable

**Appendix 5**

**Service Model Providers by Respective Systematic Review**

| **Service Model Providers** | **Reference** | **Total** |
| --- | --- | --- |
| Nurses | ^16,89,18,96,74,69,70,83,54,94,59,42,51,73,17,91,47,88,40,99,93,92,80,46,97,14,48,81,53,64,49,63,101,86,66,87,60,43,104,68,44,57,77,76,50,75,55,84,52^ | 49 |
| Physicians | ^16,89,56,18,96,74,70,83,54,94,59,42,51,73,17,91,47,88,40,52,46,80,97,14,48,81,53,64,49,66,60,104,68,57,77,65,75,50,55,84^ | 40 |
| Social workers | ^18,67,69,94,59,42,51,73,17,91,40,92,46,48,81,53,64,66,60,43,68,44,57,77,50,75,55,84,103^ | 29 |
| Physiotherapists | ^18,70,83,73,47,40,53,87,60,103,104,57,55,84^ | 14 |
| Volunteers | ^42,51,91,46,41^ | 5 |
| Home carers/home health aides | ^87,40^ | 2 |
| Formal caregivers | ^68^ | 1 |
| Other professionals^a^ | ^18,56,70,59,42,51,73,91,47,40,99,80,97,14,81,53,64,49,63,86,66,60,103,104,68,57,77,75,55,84^ | 30 |

^a^ Other professionals can include one or more of the following: occupational therapists, nutritionists and/or dieticians, pharmacists, physician assistants, nurses aides and/or nursing assistants / nursing orderlies, personal-care attendants, psychologists, chaplains and/or spiritual counselors, dentists, audiologists, rehabilitation staff, podiatrists, opticians, health visitors, health educators, care managers, nursing-care home staff, art therapists, meditation instructors, programmers, and research assistants.

**Appendix 6**

**Target Outcomes Reported in the Systematic Reviews**

| **Quality of Life** | **References** | **Total** |
| --- | --- | --- |
| Quality of life | ^16-18,36,38,40-42,46-48,50,54,55,58,59,61-64,67,68,70,71,73-77,79,80,83-86,88,89,91-94,99,103^ | 43 |
| Psychiatric symptoms, including depression anxiety and mood | ^14,17,18,36,40,41,44,53,55,59,63,64,67,70,71,80,83-85,88,92,103^ | 23 |
| Pain | ^14,16,17,36,39,42,46,48,61,62,64,67,79,82,85,86,89^ | 17 |
| Symptom control / management / burden of multiple symptoms (physical and psychological) | ^17,36,38,46,58,60-65,72,79,85,88,92^ | 16 |
| Psychological and spiritual well-being | ^17,44,63,64,80,88^ | 6 |
| Empowerment | ^67,81,83,85,91^ | 5 |
| Dyspnea | ^16,67,89^ | 3 |
| Patient morale | ^74,87^ | 2 |
| Well-being | ^74,91^ | 2 |
| Fatigue | ^67^ | 1 |
| Insight | ^63^ | 1 |
| Patients’ perception of health | ^74^ | 1 |
| **Function** | | |
| Function | ^16-18,44,47,48,51-53,57,60,63,65,68,70,71,73,74,76,81,83,88-90,97^ | 25 |
| ADLs and/or impairment | ^16,18,49,52,55,57,67,74,78,83,87^ | 11 |
| Cognitive function | ^18,44,47,52,55,63,70,84^ | 8 |
| Falls | ^18,53,87,99^ | 4 |
| Mobility | ^18,57,87^ | 3 |
| Social participation | ^57,70,74^ | 3 |
| Social support | ^67,74^ | 2 |
| Exercise performance | ^83^ | 1 |
| Frailty | ^78^ | 1 |
| **Dignified End-of-Life Care** | | |
| Patient’s and caregiver’s satisfaction with care | ^14,17,18,36,38,40,42,44,46,48,50,58,60-64,67,71,74-77,79-82,84-86,90,96,98,100,101,103,104^ | 37 |
| Living will, advanced directive, power of attorney, do-not-resuscitate order | ^14,16,75,83,90,95^ | 6 |
| Quality of care | ^18,67,70,90,101^ | 5 |
| Communication/interaction | ^67,70,90^ | 3 |
| Goals-of-care discussions documented | ^50,90^ | 2 |
| Preparation for end of life | ^63^ | 1 |
| Quality of death | ^38^ | 1 |
| **Use of Health Services** | | |
| Length of hospital stay | ^14,16-18,38,43,52,53,58,67,69,73-75,79,81,83,85,86,90,94,95,97^ | 23 |
| Readmission | ^16-18,47,50,52,53,55,67,74,76,83,90,94,95,97,100,104^ | 18 |
| Hospital admission | ^14,17,43,54,58,60,65,67,69,73,75,81-83,100^ | 15 |
| Place of death | ^14,36,38,39,46,48,50,58,60,64,75,89^ | 12 |
| Visits to emergency department | ^17,43,58,65,67,69,76,79,82,83,87,90^ | 12 |
| Use of and referral to hospice | ^14,17,39,40,50,75,83^ | 7 |
| Use of medication | ^14,56,67,70,73,84^ | 6 |
| Place of residence | ^18,43,47,48,52,87^ | 6 |
| Discharge location | ^18,53,90,95,97^ | 5 |
| Use of ICU | ^58,65,79,86^ | 4 |
| Medication-related problems | ^16,63,99,104^ | 4 |
| Use of burdensome interventions | ^63,90^ | 2 |
| Admission to nursing home | ^51,69^ | 2 |
| Use of nonpalliative services | ^40,95^ | 2 |
| Delay in diagnosis and/or treatment | ^60,104^ | 2 |
| Length of stay in hospice | ^65,83^ | 2 |
| Days spent at home | ^18^ | 1 |
| Referral to community services and/or use of services after discharge | ^74^ | 1 |
| Discharge rate | ^67^ | 1 |
| Total length of time in palliative care | ^38^ | 1 |
| Length of nursing home stay, use of formal and/or informal community services | ^43^ | 1 |
| Use of outpatient and/or inpatient services | ^71^ | 1 |
| Unmet service needs | ^44^ | 1 |
| Adverse health outcomes | ^104^ | 1 |
| **Costs** | | |
| Health-care costs | ^16,17,36,40,43,45,46,50,53,60,63-65,67,69-71,74,82,84-86,90,97,101^ | 25 |
| Nursing-home costs | ^67,74,94^ | 3 |
| Cost-effectiveness | ^70,82^ | 2 |
| Quality-adjusted life years and/or days | ^70,82^ | 2 |
| Self-reported health-care costs | ^67^ | 1 |
| Costs of caregiver | ^60^ | 1 |
| **Survival** | | |
| Survival | ^17,18,40,44,47,50-56,59,61,65,67,68,71-74,82,89,90,94,95,97,104^ | 28 |
